# Supplementary material for: Manipulation of Spray-Drying Conditions to Develop an Inhalable Ivermectin Dry Powder
Source: Pharmaceutics. 2022 Jul 8;14(7):1432. doi: 10.3390/pharmaceutics14071432 (PMC9325229; doi:10.3390/pharmaceutics14071432)
Supplement: Supplementary file 1 [file pharmaceutics-14-01432-s001.zip › pharmaceutics-1767489-supplementary.pdf]

pharmaceutics

Supplementary Materials: Manipulation of Spray-Drying Conditions to Develop an Inhalable Ivermectin Dry Powder

Tushar Saha[1], Shubhra Sinha[2], Rhodri Harfoot[2], Miguel E. Quiñones-Mateu[2,3] and Shyamal C. Das[1]*

[1] School of Pharmacy, University of Otago, Dunedin 9054, New Zealand.
[2] Department of Microbiology and Immunology, School of Biomedical Sciences, University of Otago, Dunedin 9054, New Zealand.
[3] Webster Centre for Infectious Diseases, University of Otago, Dunedin 9054, New Zealand.
* Correspondence: shyamal.das@otago.ac.nz; Tel.: +64 3 479 4262

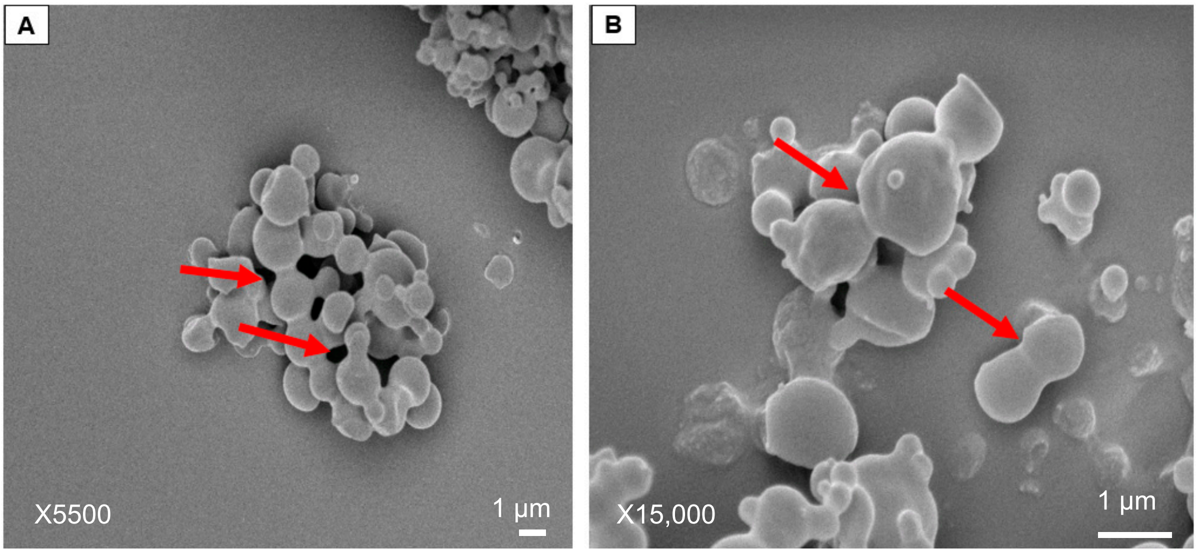

Figure S1. Fused ivermectin dry powder after spray drying at (A) 120°C and (B) 140°C. (Arrows indicating the fused particles).

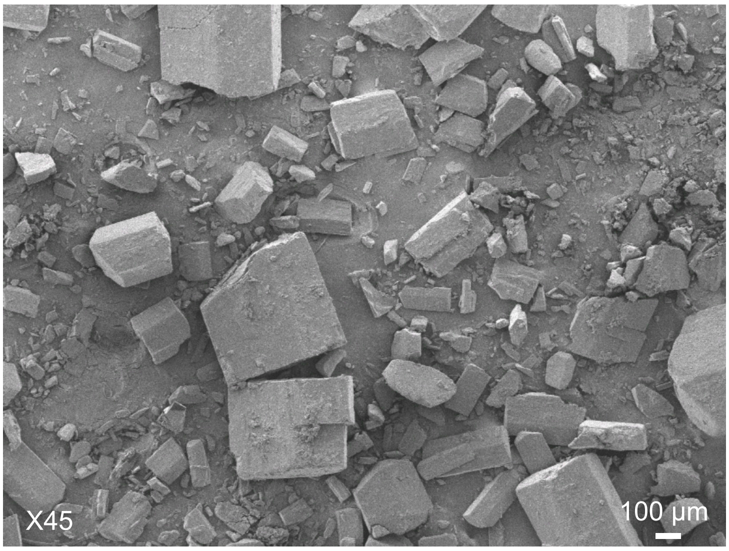

Figure S2. Powder morphology of ivermectin raw material.

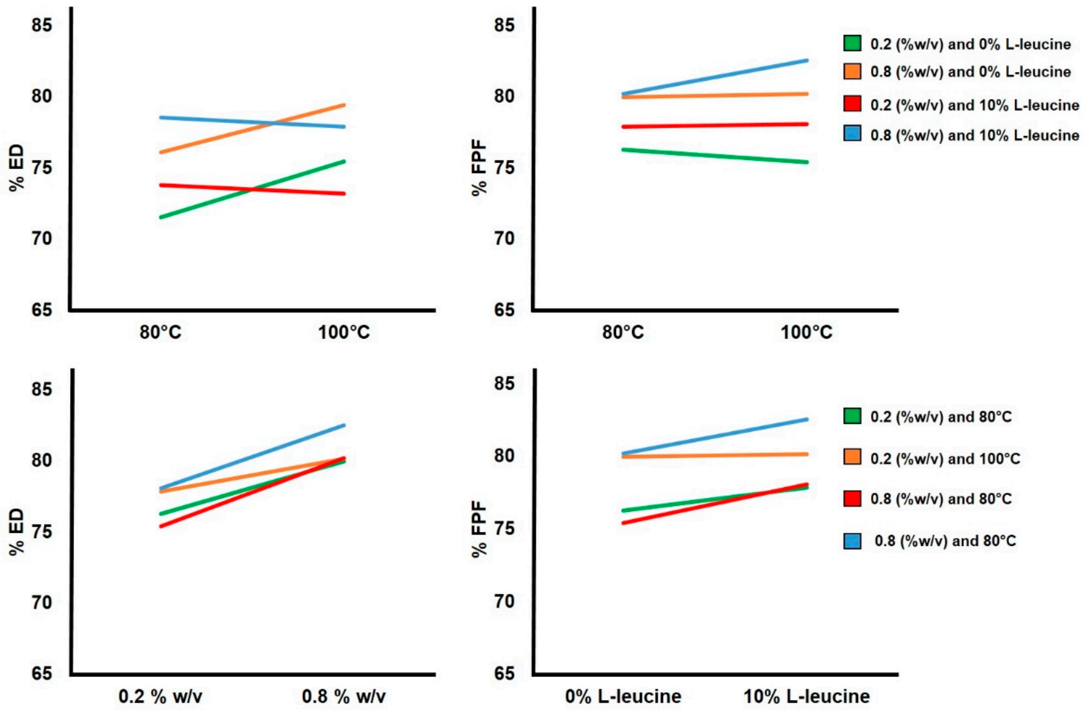

Figure S3. Influence of inlet temperature and L-leucine on emitted dose (ED) and fine particle fraction (FPF).

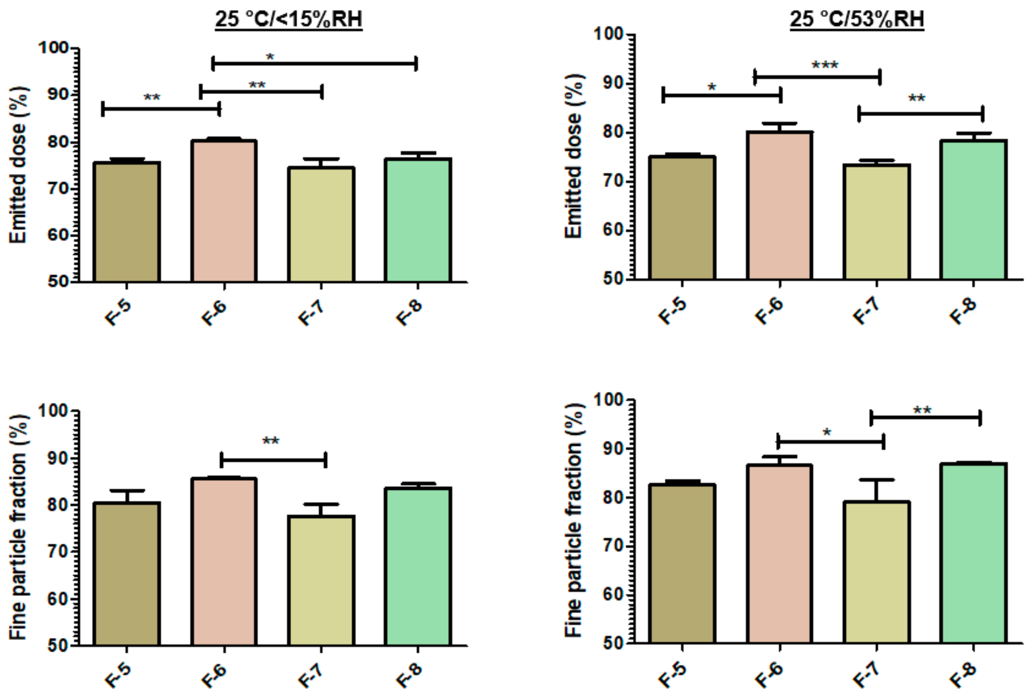

Figure S4. In vitro aerosolization of prepared ivermectin dry powder after 28 days stability study (25°C/<15%RH and 25°C/53%RH). The parameters of stated formulations are - (F-5) 0.2% ($^{w}/_{v}$) feed concentration, 100 °C and 0% L-leucine (F-6) 0.8% ($^{w}/_{v}$) feed concentration, 100 °C and 0% L-leucine (F-7) 0.2% ($^{w}/_{v}$) feed concentration, 100 °C and 10% L-leucine (F-8) 0.8% ($^{w}/_{v}$) feed concentration, 100 °C and 10% L-leucine. (* indicating $p < 0.05$, ** $p < 0.01$, *** $p < 0.001$).

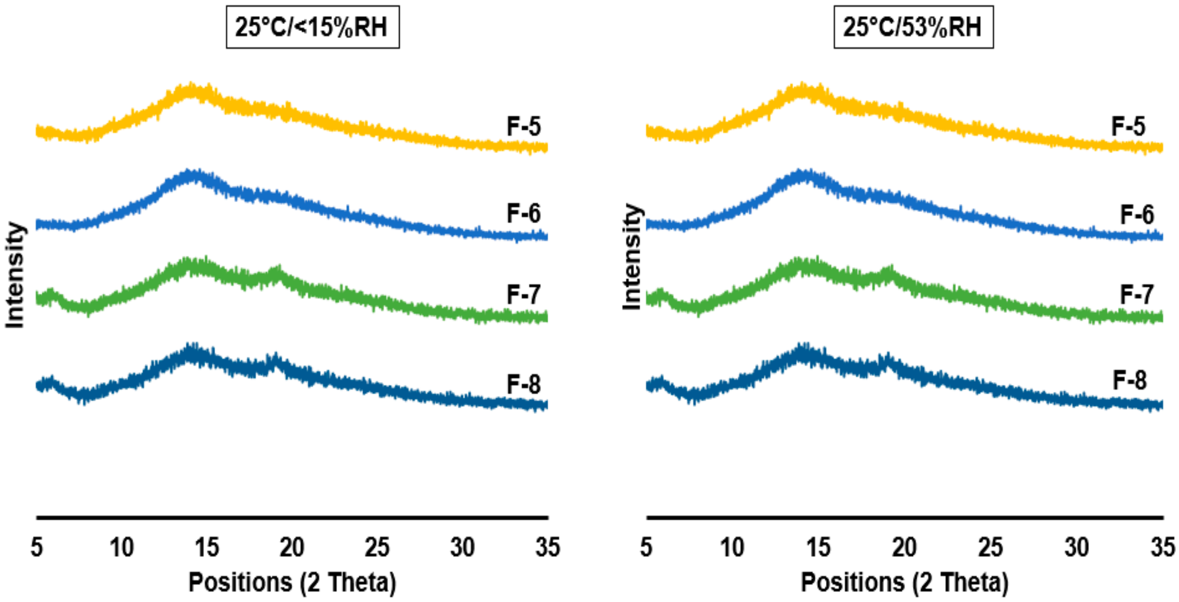

Figure S5. X-ray diffractograms of spray-dried formulations after 28 days stability study (25°C/<15%RH and 25°C/53%RH). The formulations were prepared at different conditions - (F-5) 0.2% (w/v) feed concentration, 100 °C and 0% L-leucine (F-6) 0.8% (w/v) feed concentration, 100 °C and 0% L-leucine (F-7) 0.2% (w/v) feed concentration, 100 °C and 10% L-leucine (F-8) 0.8% (w/v) feed concentration, 100 °C and 10% L-leucine. No peaks in the formulations indicating the amorphous nature.

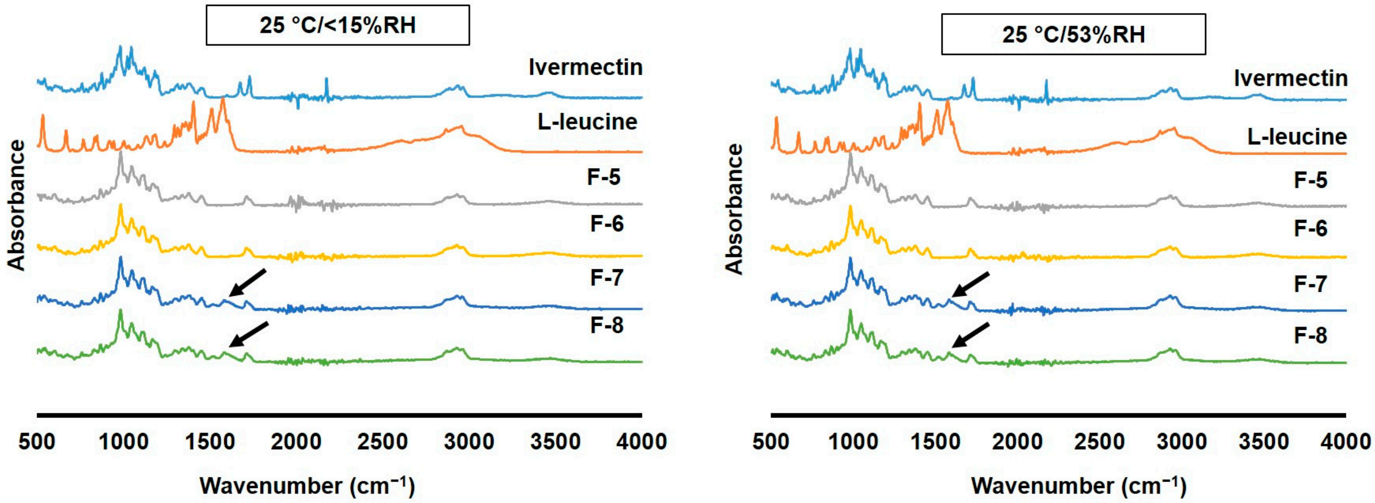

Figure S6. ATR-FTIR spectra of supplied ivermectin, L-leucine and spray dried formulations after 28 days stability study (25°C/<15%RH and 25°C/53%RH). The formulations were prepared at different conditions - (F-5) 0.2% (w/v) feed concentration, 100 °C and 0% L-leucine (F-6) 0.8% (w/v) feed concentration, 100 °C and 0% L-leucine (F-7) 0.2% (w/v) feed concentration, 100 °C and 10% L-leucine (F-8) 0.8% (w/v) feed concentration, 100 °C and 10% L-leucine. (Arrows indicating L-leucine peak).

Table S1. In vitro aerosolization behaviour of prepared ivermectin dry powder.

Formulation	Loaded mass (mg)	Mass recovery (%)	FPD (mg)	%ED	%FPF
F-1	18.3 ± 0.2	102.2 ± 2.9	10.3 ± 0.2	71.5 ± 0.7	76.3 ± 1.8
F-2	18.2 ± 0.1	102.3 ± 0.2	11.3 ± 0.4	76.1 ± 4.4	79.9 ± 2.0
F-3	19.9 ± 0.9	93.8 ± 0.6	10.8 ± 0.6	73.8 ± 2.2	77.9 ± 3.1
F-4	19.8 ± 0.6	97.5 ± 1.2	12.1 ± 0.6	78.5 ± 1.3	80.2 ± 1.0
F-5	19.4 ± 0.3	101.1 ± 3.5	11.3 ± 0.8	75.4 ± 1.1	75.4 ± 2.1
F-6	18.9 ± 0.2	97.7 ± 1.1	11.9 ± 0.2	79.4 ± 1.3	80.2 ± 1.2
F-7	18.1 ± 0.1	97.6 ± 0.9	10.2 ± 0.1	73.2 ± 0.3	78.1 ± 0.2
F-8	19.5 ± 0.1	99.5 ± 1.4	12.4 ± 0.2	77.9 ± 0.5	82.5 ± 1.4

FPD = Fine particle dose, ED = Emitted dose, FPF = Fine particle fraction

Table S2. In vitro aerosolization behaviour of ivermectin dry powder after 28 days stability study (25°C/<15%RH and 25°C/53%RH).

Formulation	25 °C/<15% RH		25 °C/<53% RH	
	% ED	%FPF	% ED	%FPF
F-5	75.6 ± 0.7	80.4 ± 2.2	75.0 ± 0.5	82.2 ± 0.7
F-6	80.2 ± 0.4	85.5 ± 0.2	80.0 ± 1.5	86.6 ± 1.4
F-7	74.5 ± 1.6	77.6 ± 2.1	73.4 ± 0.7	79.1 ± 3.7
F-8	76.5 ± 1.0	83.6 ± 0.7	78.4 ± 1.2	86.9 ± 0.3

ED = Emitted dose, FPF = Fine particle fraction.

Table S3. The water content of ivermectin dry powder after 28 days stability study (25°C/<15%RH and 25°C/53%RH).

Formulation	Water content	
	25 °C/<15% RH	25 °C/<53% RH
F-5	0.5 ± 0.1	0.6 ± 0.1
F-6	0.4 ± 0.1	0.5 ± 0.1
F-7	0.3 ± 0.1	0.5 ± 0.1
F-8	0.4 ± 0.1	0.6 ± 0.1
